# CCDC28A deficiency causes sperm head defects, reduced sperm motility and male infertility in mice

**DOI:** 10.1007/s00018-024-05184-5

**Published:** 2024-04-10

**Authors:** Hongbin Zhou, Zhihua Zhang, Ronggui Qu, Hongying Zhu, Yuxi Luo, Qun Li, Jian Mu, Ran Yu, Yang Zeng, Biaobang Chen, Qing Sang, Lei Wang

**Affiliations:** 1 Institute of Pediatrics, The Institutes of Biomedical Sciences, The State Key Laboratory of Genetic Engineering, School of Life Sciences, Children’s Hospital of Fudan University, Fudan University, Shanghai, 200032 China; 2 NHC Key Lab of Reproduction Regulation (Shanghai Institute for Biomedical and Pharmaceutical Technologies), Fudan University, Shanghai, 200032 China

**Keywords:** CCDC28A, Knockout, Sperm head defects, Motility, Male infertility

## Abstract

Mature spermatozoa with normal morphology and motility are essential for male reproduction. The epididymis has an important role in the proper maturation and function of spermatozoa for fertilization. However, factors related to the processes involved in spermatozoa modifications are still unclear. Here we demonstrated that CCDC28A, a member of the CCDC family proteins, is highly expressed in testes and the CCDC28A deletion leads to male infertility. We found CCDC28A deletion had a mild effect on spermatogenesis. And epididymal sperm collected from *Ccdc28a*^−/−^ mice showed bent sperm heads, acrosomal defects, reduced motility and decreased in vitro fertilization competence whereas their axoneme, outer dense fibers, and fibrous sheath were all normal. Furthermore, we found that CCDC28A interacted with sperm acrosome membrane-associated protein 1 (SPACA1) and glycogen synthase kinase 3a (GSK3A), and deficiencies in both proteins in mice led to bent heads and abnormal acrosomes, respectively. Altogether, our results reveal the essential role of CCDC28A in regulating sperm morphology and motility and suggesting a potential marker for male infertility.

**Supplementary Information:**

The online version contains supplementary material available at 10.1007/s00018-024-05184-5.

## Introduction

Mature spermatozoa with normal morphology and motility are a prerequisite for male reproduction. In mammals, spermatozoa are generated in the male testis and undergo maturation in the epididymis. Spermatogenesis is a complex developmental process including proliferation of undifferentiated spermatogonia, the meiotic differentiation of spermatogonia to produce haploid spermatids, the transformation of haploid round spermatids into elongated spermatids, and the release of elongated spermatids into the epididymis tract [1]. The spermatozoa undergo significant modifications in terms of their biochemical and morphological properties during their transit through the epididymis from the caput to the cauda epididymis, and these transformations give them the ability to move progressively and to recognize and fertilize oocytes [2–4]. Successful spermatogenesis requires the accurate spatio-temporal regulation of a host of genes, especially testis-specific genes. It has been reported that over 2000 genes are involved in the process of spermatogenesis [5], and most of genes perform functions and their underlying molecular mechanisms remain largely unclear and require further elucidation. Terminal modifications of spermatozoa from testis occur during their transit through the long epididymal tubule and some of these genes are known to play a role in sperm maturation. For instance, TMEM225, GSK3A and SPEM1 are expressed in the testis might not cause spermatogenic aberrations in the testis, but they are critical for sperm maturation and male fertility [6–8].

*Ccdc28a* (coiled-coil domain containing 28A) is a gene that shows the highest expression in male testis compared to all other tissues in the adult mouse (https://www.proteinatlas.org). However, the physiological function of CCDC28A and whether it plays a role during spermatogenesis is unknown. Previous studies of CCDC28A were mainly focused on tumors. For example, *CCDC28A* and *NUP98* can fuse via translocation to form a new fusion gene that encodes a CCDC28A-NUP98 fusion protein, which promotes the proliferation of bone marrow cells and leads to malignant myeloproliferative tumors [9]. In addition, CCDC28A exhibits antigenic responses in low-grade gliomas, suggesting that it might serve as a biomarker in the early stages of disease [10]. However, there are currently no studies on the relationship between CCDC28A and male fertility.

To explore the function of CCDC28A and its possible role during male reproduction, we constructed *Ccdc28a* knockout mice using CRISPR/Cas9 genome editing technology. We found that *Ccdc28a* knockout male mice were sterile due to severe sperm malformations in the epididymis, including disrupted acrosome assembly and bent heads, whereas spermatogenesis in the testis was subtly affected. In addition, mass spectrometer-based proteomic analysis, western blotting, and co-immunoprecipitation assays were performed to demonstrate that CCDC28A is an interacting partner of sperm acrosome membrane-associated protein 1 (SPACA1) and glycogen synthase kinase 3a (GSK3A). These findings illustrate the essential role of CCDC28A in regulating sperm morphology and motility.

## Results

### *Ccdc28a* was highly expressed in male testis and was required for male fertility

**Fig. 1:**
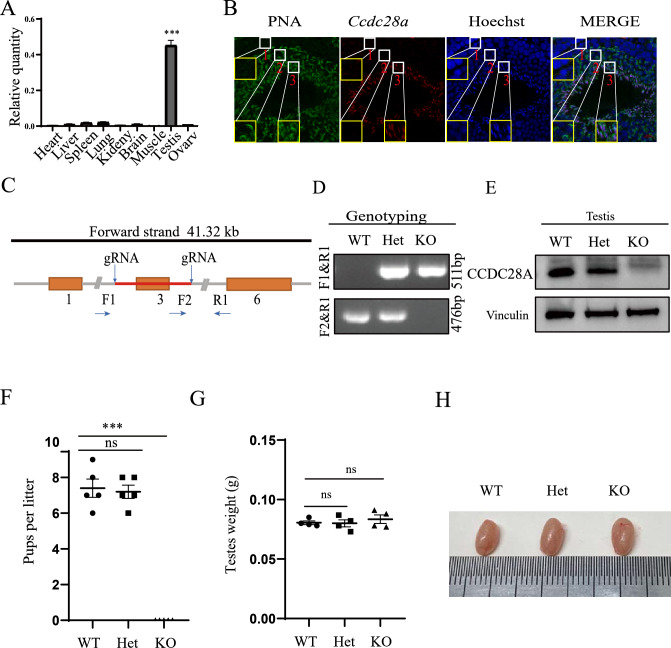
CCDC28A was essential for male fertility. **A** RT-PCR analysis of *Ccdc28a* expression in various adult mouse tissues. *Gapdh* mRNA was used as the internal control. One-way ANOVA, ***P ≤ 0.001. **B** In situ hybridization for *Ccdc28a* mRNA on epididymis sections of sexually mature mice. Representative images of mouse epididymis epididymis sections stained for PNA (green), *Ccdc28a* mRNA (red), Hoechst (blue). 1: Spermatocytes (SCs), 2: Round spermatids, 3: Elongating spermatids. **C** Schematic diagram of the CRISPR/Cas9 targeting strategy to generate *Ccdc28a* knockout mice. sgRNAs were designed to specifically direct Cas9 to sites flanking exon 3 of *Ccdc28a*, leading to a frameshift mutation and premature translation termination. **D** PCR genotyping of tail-derived genomic DNA of *Ccdc28a*^+/+^, *Ccdc28a*^+/–^, and *Ccdc28a*^–/–^mice. **E** Western blot analysis of *Ccdc28a* expression in the testis of *Ccdc28a*^+/+^, *Ccdc28a*^+/–^, and *Ccdc28a*^–/–^ mice. Vinculin was used as the internal control. The results are presented as the mean ± SEM. ns, non-significant. **F** The average number of pups per litter of *Ccdc28a*^+/+^, *Ccdc28a*^+/–^, and *Ccdc28a*^–/–^ male mice after mating with wild type female mice. *Ccdc28a*^–/–^ male mice were sterile. Data were analyzed by one-way ANOVA, ***P ≤ 0.001. **G** *Ccdc28a*^+/+^, *Ccdc28a*^+/–^, and *Ccdc28a*^–/–^ testis weights at 8–10 weeks of age. Data were analyzed by one-way ANOVA. ns, non-significant. N = 3. **H** Gross morphology of representative testes from *Ccdc28a*^+/+^, *Ccdc28a*^+/–^, and *Ccdc28a*^–/–^ mice. *WT* wildtype, *Het* heterozygote, *KO* knockout

To investigate the role of *Ccdc28a* in male fertility, we first detected the expression pattern of *Ccdc28a* in different tissues using qRT-PCR. In adult mice, *Ccdc28a* was highly expressed in the testis and there was little or no *Ccdc28a* expression in other tissues (Fig. 1A), which suggests a role in the process of male fertility. Besides that, in situ hybridization was done on mouse testicular and epididymal sections by using a *Ccdc28a* antisense probe, which showed that *Ccdc28a* expressed in elongating spermatids but not spermatogonia, spermatocytes and epididymal epitheliums (Fig. 1B, S1). To further evaluate the role of *Ccdc28a* in male fertility, we generated *Ccdc28a*^–/–^ mice using CRISPR/Cas9 genome editing technology (Fig. 1C). PCR analysis of genomic DNA revealed that the *Ccdc28a* gene was successfully disrupted in targeted mice (Fig. 1D). Western blotting analysis further confirmed that these mice failed to produce functional CCDC28A protein (Fig. 1E). A pair breeding assay showed that *Ccdc28a*^–/–^ males mating with wild type females did not deliver any offspring (Fig. 1F), while *Ccdc28a*^–/–^ females showed no fertility abnormalities (data not shown). The testis weights showed no obvious differences between the *Ccdc28a*^–/–^ and *Ccdc28a*^+/+^ mice (Fig. 1G). Further studies showed that the morphology of testes from *Ccdc28a*^–/–^ mice were not affected (Fig. 1H). These results suggest that *Ccdc28a* was indispensable for male infertility.

### CCDC28A disruption impaired sperm motility and morphology

**Fig. 2:**
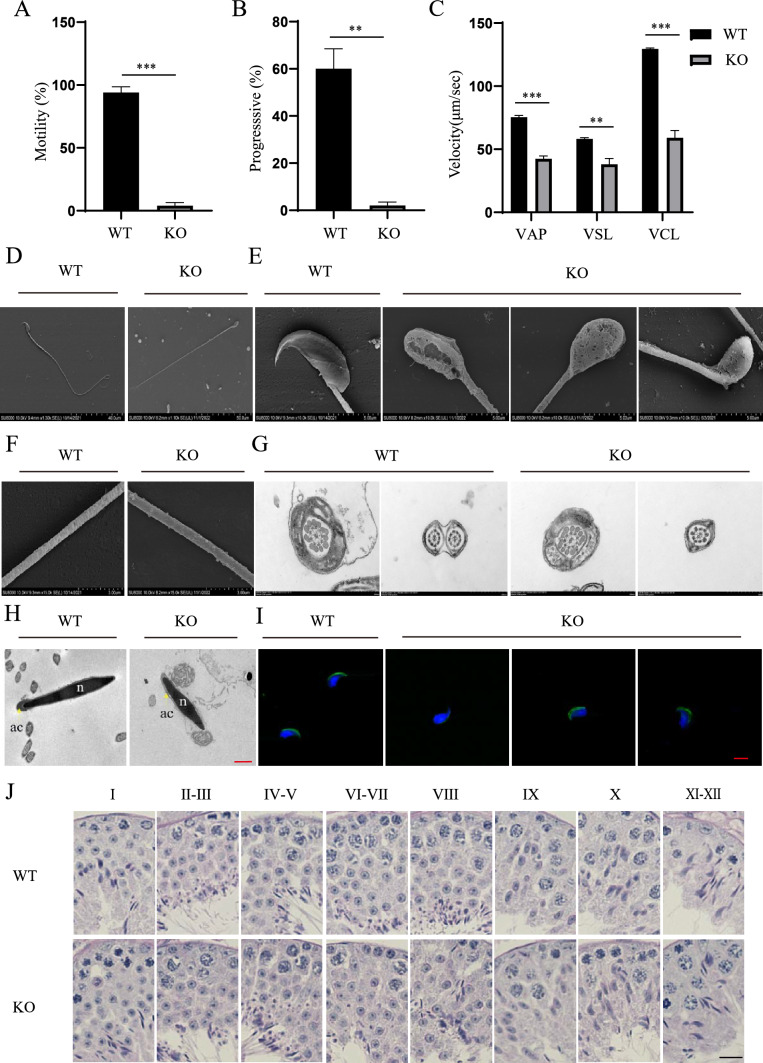
CCDC28A disruption impaired sperm motility and morphology. **A** CASA measurements of the percentage of epididymal sperm that were motile. Data are presented as mean ± SEM. Student’s t-test; ***P < 0.001. N = 3. **B** CASA measurements of progressive sperm motility. Data are presented as the mean ± SEM. Student’s t-test, ***P < 0.001. N = 3. VAP, average path velocity; VSL, straight line velocity; VCL, curvilinear velocity. **C** The motility parameters are expressed as significant differences compared to control male mice. Data are presented as the mean ± SEM. Student’s t-test, **P < 0.01; *** *P* < 0.001. N = 3. **D** Scanning electron microscopy analysis of the overall morphology of cauda epididymal sperm from *Ccdc28a*^+/+^ and *Ccdc28a*^–/–^ mice. **E** The head morphology of cauda epididymal sperm. Sperm from *Ccdc28a*^–/–^ mice showed abnormal head shapes and bent heads. **F** The mitochondrial sheath along the midpiece of cauda epididymal sperm. *Ccdc28a* loss did not appear to affect the mitochondrial sheath. **G** Cross sections of sperm flagella from the cauda epididymis of *Ccdc28a*^+/+^ and *Ccdc28a*^–/–^ mice. **H** TEM analysis showing detachment of the acrosome (ac, yellow arrows) from the sperm nuclei (n) in *Ccdc28a*^–/–^ sperm. Scale bar = 1 μm. **I** Representative images of cauda epididymal sperm from *Ccdc28a*^+/+^ and *Ccdc28a*^–/–^ mice stained for PNA (green), and DNA was counterstained with Hoechst (blue). Scale bar = 5 μm. **J** PAS-stained sections of seminiferous tubules from *Ccdc28a*^+/+^ and *Ccdc28a*^–/–^ mice at stages IX–XII of spermatogenesis. Scale bar = 5 mm

To explore the underlying reasons for the infertility of *Ccdc28a*^−/−^ male mice, sperm from *Ccdc28a*^+/+^ and *Ccdc28a*^–/–^ mice were sampled for motility and morphology analysis. The motility assay showed that *Ccdc28a* knockout significantly decreased the percentage of motile sperm and the progressive motility of sperm (Fig. 2A, B). We further evaluated the sperm motility parameters defining the speed of sperm motion using CASA (Computer-aided sperm analysis) in *Ccdc28a*^+/+^ and *Ccdc28a*^–/–^ mice. The average path velocity, straight line velocity, and curvilinear velocity were decreased in knockout sperm compared to controls (Fig. 2C). Scanning electron microscopy further confirmed that the sperm from the *Ccdc28a*^–/–^ mice showed obvious sperm head defects including bent heads, microcephaly, and irregular shapes (Fig. 2D, E), while the sperm flagellum from both *Ccdc28a*^+/+^ and *Ccdc28a*^–/–^ mice had normal morphology (Fig. 2D, F). We also used transmission electron microscopy (TEM) to analyze the flagellum ultrastructure in CCDC28A-deficient sperm. CCDC28A loss did not affect the axoneme, outer dense fibers, or fibrous sheath (Fig. 2G).

Acrosome integrity has been reported to be important for sperm head formation and successful fertilization [11], so we evaluated the sperm acrosome of *Ccdc28a*^+/+^ and *Ccdc28a*^–/–^ male mice by TEM and immunofluorescence analysis. The sperm acrosome from *Ccdc28a*^+/+^ mice had a single cap-like structure that covered the anterior portion of the hook-shaped head and was tightly attached to the nucleus. The *Ccdc28a*-null sperm exhibited multiple forms of acrosome imperfections such as detachment of the acrosome from the nucleus, as confirmed by TEM observation (Fig. 2H). To confirm this observation, examination of the PNA (peanut agglutinin) staining in control and knockout mice showed that the sperm from knockout mice had abnormal acrosome structures, such as acrosome detachment from the sperm nucleus, irregular-shaped acrosomes, and fragmented acrosomes (Fig. 2I). These results suggested that the deletion of *Ccdc28a* led to low sperm motility and abnormal sperm morphology.

### *Ccdc28a* deletion show mild effect on spermatogenesis and subsequent loss of acrosome and sperm head coiling during the epididymal transit

**Fig. 3:**
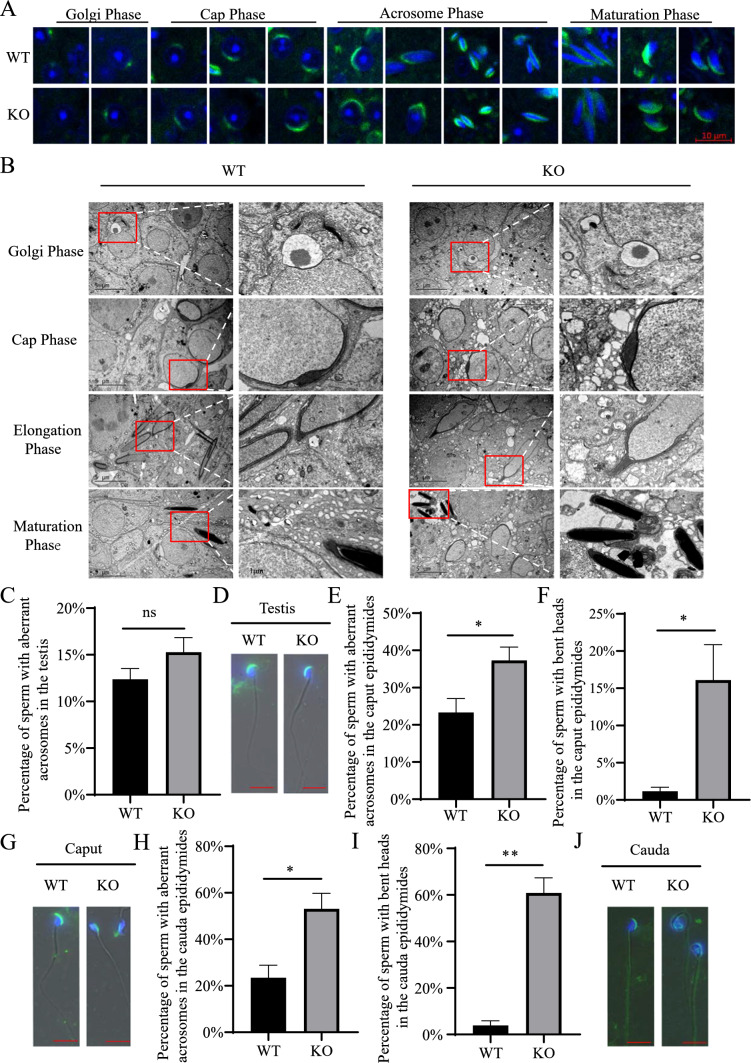
Spermatogenesis was mildly disrupted in the testes of CCDC28A deficient mice. **A** Acrosome and proacrosomal vesicle staining using PNA-FITC. Spermiogenesis can be divided through PNA staining into four distinct phases: the Golgi, the cap, the acrosome, and the maturation phase. Nuclei are stained blue (Hoechst), acrosomes green (lectin-PNA). **B** TEM showed seemingly similar acrosome biogenesis in the testes of *Ccdc28a*^+/+^ and *Ccdc28a*^−/−^ mice during the Golgi, Cap, Elongation, and Maturation phases. Scale bar = 1 μm. **C** Quantification of spermatozoa with aberrant acrosomes from 8-week-old *Ccdc28a*^+/+^and *Ccdc28a*^−/−^ mice (n = 3). Student's t test; ns, non-significant. **D** representative images of spermatozoa from *Ccdc28a*^+/+^and *Ccdc28a*^−/−^ mice testes stained for PNA (green). DNA was counterstained with Hoechst (blue). Scale bars = 5 μm. **E**, **F** Quantification of spermatozoa with aberrant acrosomes or bent heads from caput epididymis (n = 3). Student’s t test; *, P < 0.05. **G** Representative images of spermatozoa from *Ccdc28a*^+/+^and *Ccdc28a*^−/−^ mice caput epididymis stained for PNA (green). DNA was counterstained with Hoechst (blue). **H**, **I** Quantification of spermatozoa with aberrant acrosomes or bent heads from cauda epididymis (n = 3). Student’s t test; *, P < 0.05, **, P < 0.01. **J** Representative images of spermatozoa from *Ccdc28a*^+/+^and *Ccdc28a*^−/−^ mice caput epididymis stained for PNA (green). DNA was counterstained with Hoechst (blue), Scale bars = 5 μm. Caput (Caput epididymis), Cauda (Cauda epididymis)

To determine the developmental stage at which defects occurred in CCDC28A deficient mice, we analyzed testis histology. Testis sections obtained from adult WT and *Ccdc28a*^–/–^ mice were stained with periodic acid Schiff (PAS), and this showed that the Sertoli, spermatogonia, spermatocytes, round spermatids, and elongated spermatids of *Ccdc28a*^–/–^ mice were all similar to those of wild-type males (Fig. 2J). The PNA staining of the testis sections show that the main stages of spermatogenesis had no obvious difference including the acrosomal integrity between WT and KO mice (Fig. 3A). To further confirmed this, we carefully evaluated the TEM of testis tissues and found a relative loosened acroplaxome structure in *Ccdc28a*-KO spermatids at the elongation/maturation acrosome phase of acrosome formation (Fig. 3B). These evidences indicated that *Ccdc28a* deletion had relative weak effect on spermatogenesis in the testis. In addition, we performed the PNA staining of the sperm from the testis and epididymis and counted the ratio of the sperm abnormalities. The result showed that the percentage of defective sperm from the WT and KO testis are comparable (Fig. 3C, D). However, the percentage of sperms with acrosomal defects and bent heads collected from the caput and cauda epididymis of *Ccdc28a* KO mice was significantly higher than that from the WT mice (Fig. 3E–J). We supposed that the *Ccdc28a* deletion had mild effect on the acrosomal structure of the spermatogenesis in the testis and more severe defective sperm occurred due to mechanical shear during the passage of sperm through the epididymis and epididymal storage. Besides, a terminal deoxynucleotidyl transferase-mediated dUTP nick end labeling (TUNEL) assay of testes sections showed apoptotic features of spermatids, but there were no appreciable differences between the *Ccdc28a*^+/+^ and *Ccdc28a*^–/–^ mice (Figure S2).

### CCDC28A deficiency affects SPACA1 and GSK3A protein levels in epididymal sperm

**Fig. 4:**
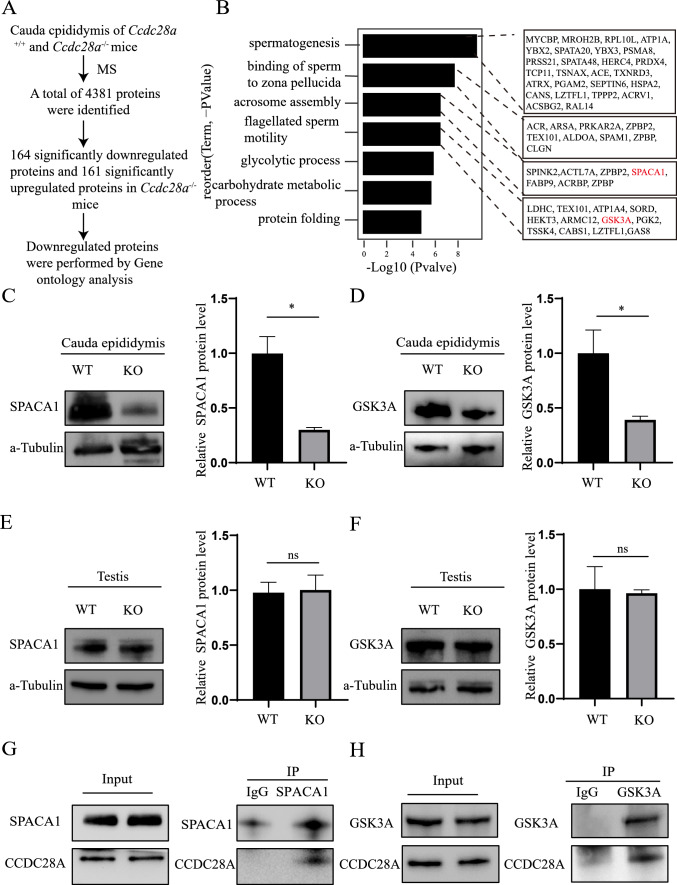
CCDC28A coupled SPACA1 and GSK3A in vivo. **A** Downregulated proteins were subjected to GO analysis. **B** GO analysis of proteins downregulated in the cauda epididymis of *Ccdc28a*^−/−^ mice. GO analysis showed that CCDC28A depletion resulted in aberrantly expressed proteins related to spermatogenesis, binding of sperm to the zona pellucida, acrosome assembly, and flagellated sperm motility. **C** Western blotting showing the protein level of SPACA1 in the cauda epididymis of *Ccdc28a*^+/+^ and *Ccdc28a*^–/–^ mice, with a-Tubulin as the loading control. Two-tailed Student’s t-test, *P ≤ 0.05. **D** Western blotting showing the protein level of GSK3A in the cauda epididymis of *Ccdc28a*^+/+^ and *Ccdc28a*^–/–^ mice, with a-Tubulin as the loading control. Two-tailed Student’s t-test, *P ≤ 0.05. **E** The SPACA1 protein level in testes from wild-type and knockout mice showed no obvious differences. a-Tubulin was used as the loading control. Two-tailed Student’s t-test; ns, non-significant. **F** The GSK3A protein level of testes in wild-type and knockout mice showed no obvious differences. a-Tubulin was used as the loading control. Two-tailed Student’s t-test; ns, non-significant. **G** Cauda epididymal extracts were subjected to immunoprecipitation with anti-SPACA1 antibody with rabbit IgG as the negative control. **H** Testis extract was subjected to immunoprecipitation with anti-GSK3A antibody with rabbit IgG as the negative control

To elucidate the molecular mechanism for the phenotypes caused by *Ccdc28a* deletion, protein expression profiles in the cauda epididymis of adult *Ccdc28a*^+/+^and *Ccdc28a*^–/–^ mice were assessed by mass spectrometry (MS) analysis. A total of 4381 proteins were identified (Fig. 4A), and among them 164 proteins were significantly downregulated and 161 proteins were significantly upregulated in *Ccdc28a*^–/–^ mice (Supplementary Table 1). Gene ontology (GO) analysis of downregulated proteins showed that these proteins were enriched in spermatogenesis [YBX2 (Y box protein 2), ATP1A4 (ATPase, Na + /K + transporting, alpha 4 polypeptide), SPATA20 (spermatogenesis associated 20) and RPL10L (ribosomal protein L10-like)]; binding of sperm to the zona pellucida [ARSA (arylsulfatase A), ALDOA (aldolase A, fructose-bisphosphate), CLGN (calmegin), and SPAM1 (sperm adhesion molecule 1)]; acrosome assembly [SPINK2 (serine peptidase inhibitor, Kazal type 2), ACTL7A (actin-like 7a), ACRBP (proacrosin binding protein), and SPACA1 (sperm acrosome associated 1)]; and flagellated sperm motility [LDHC (lactate dehydrogenase C), GAS8 (growth arrest specific 8), SORD (sorbitol dehydrogenase), and GSK3A (glycogen synthase kinase 3 alpha)].

According to the phenotypic characteristics and phenotypic assays in *Ccdc28a*^−/−^ mice, we selected significantly differentially expressed proteins from the MS data associated with acrosome formation and bent heads for functional verification. It has been reported that disruption of SPACA1 leads to infertility due to abnormal sperm head shape and abnormal acrosomes in sperm, and GSK3A-deficient mice show bent sperm heads [6, 12], similar to the phenotype of *Ccdc28a*^−/−^ mice. Compared with *Ccdc28a*^+/+^ mice, decreased protein levels of SPACA1 and GSK3A in the cauda epididymis of *Ccdc28a*^−/−^ mice were confirmed by western blotting (Fig. 4C, D). Interestingly, there was no obvious difference in SPACA1 and GSK3A protein levels in the testes of *Ccdc28a*^+/+^ and *Ccdc28a*^−/−^ adult mice (Fig. 4E, F). In addition, the SPACA1 staining of testis sections showed that *Ccdc28a* deletion didn’t affected the SPACA1 localization in elongating spermatids (Fig S3). Through co‐immunoprecipitation analysis, we found that CCDC28A couples with SPACA1 and GSK3A (Fig. 4G, H). Taken together, these results suggest that deletion of mouse CCDC28A might affect sperm morphology and motility by regulating SPACA1 and GSK3A.

### The fertilization failure of ***Ccdc28a***^–/–^ male mice was rescued by ICSI

**Fig. 5:**
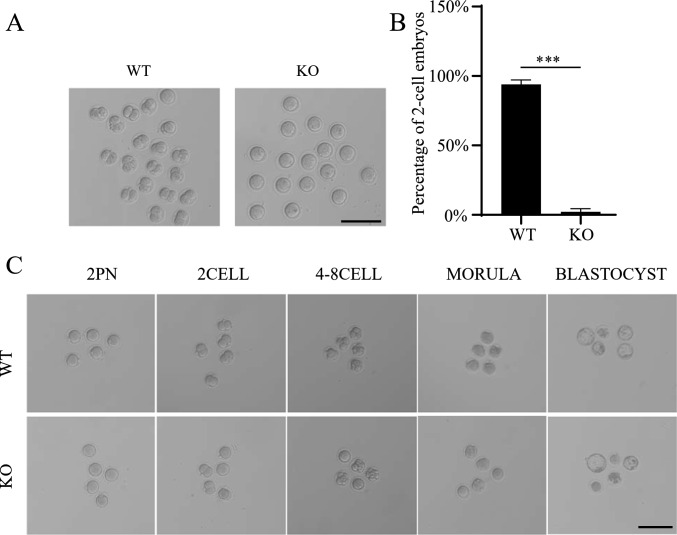
The infertile phenotype of *Ccdc28a*^*–/–*^ mice was rescued by ICSI. **A** Light micrographs showing two-cell embryos of normal oocytes fertilized with *Ccdc28a*^*–/–*^ and control sperm. **B** The two-cell embryo rates were calculated at 24 h post fertilization. Data indicate the mean ± SEM, two-tailed Student’s t-test, ***P ≤ 0.001. N = 3. Scale bar = 100 µm. **C**, Light micrographs showing early embryonic development from MII oocytes inseminated by ICSI. Heads of sperm from *Ccdc28a*^+/+^and *Ccdc28a*^–/–^ male mice were injected into MII oocytes from wild type female mice. 2PN, 2 pronuclei. Scale bar = 100 μm

The above results suggested that *Ccdc28a*^–/–^ male mice are infertile due to abnormal sperm morphology and reduced sperm motility. Because the *Ccdc28a*^–/–^ male mice are completely sterile despite normal mating behavior, we performed IVF (in vitro fertilization) using excess sperm from the cauda epididymis of *Ccdc28a*^+/+^ and *Ccdc28a*^−/−^ male mice. However, the number of two-cell embryos that formed in the *Ccdc28a*^−/−^ group was significantly decreased compared to the *Ccdc28a*^+/+^ group (Fig. 5A, B). Clinically, intracytoplasmic sperm injection (ICSI) has been confirmed as an effective way to overcome fertilization failure due to severe teratospermia [13]. We attempted to rescue the fertilization failure of *Ccdc28a*^–/–^ male mice using ICSI, and as expected sperm from *Ccdc28a*^–/–^ male mice could fertilize wild type female oocytes, forming 2-pronuclei zygotes that further developed into 2 cells, 4–8 cells, and blastocysts, which was consistent with the ICSI results in *Ccdc28a*^+/+^ mice (Fig. 5C).

## Discussion

Teratozoospermia, one of the main causes of male infertility, is characterized by malformed spermatozoa. A number of transgenic and knockout mouse models have been shown to cause sperm malformation that can affect male fertility, indicating that structural integrity is essential for proper sperm function [14, 15].

For instance, deletion of GOPC (Golgi-associated PDZ and cooled-coil motif contained), PICK1 (protein interacting with PRKCA 1), and CSNK2A2 (casein kinase 2 alpha 2) results in defects in acrosomal formation and in deformed sperm heads [16–18]. The disruption of DNAH1 (dynein axonemal heavy chain 1), CFAP65 (cilia and flagella-associated protein 65), and CFAP251 (cilia and flagella-associated protein 251) leads to multiple morphological abnormalities of the sperm flagella [19–21], and mice lacking SPEM1 (spermatid maturation protein 1) and PPP1CC2 (serine/threonine-protein phosphatase PP1-gamma catalytic subunit) have bent heads in the majority of their sperm [7, 22].

In the present study, we found that CCDC28A was essential for male fertility. Sperm from CCDC28A deficient mice had bent heads and showed clear defects in acrosome formation. Through western blotting and immunoprecipitation, we confirmed that CCDC28A might play a role in male fertility through its interaction with SPACA1 and GSK3A. SPACA1, also known as SAMP32, is located in the inner membrane of the acrosome and is an acrosomal membrane protein that can be detected at all stages of the seminiferous epithelial cycle [12]. SPACA1-deficient mice show a globozoospermia-like phenotype and infertility [12]. In addition, SPACA1 interacts with ACTL7A (which was also decreased in our MS data) to anchor the acrosome to the acroplaxome [23]. GSK3A, a serine/threonine protein kinase, is located in the acrosome and flagella of sperm and is involved in a large number of cellular processes [6]. Morphological analysis of male mice lacking GSK3A showed that bent sperm heads were present in the cauda epididymis, but not in the testis [6], which is similar to our observation. Furthermore, sperm motility and the velocity parameters of *Gsk3a* knockout mice were significantly reduced. Interestingly, *Ccdc28a* deletion had a weak effect on spermatogenesis in terms of PNA lectin staining, PAS histochemistry and TEM. We supposed that the *Ccdc28a* deletion may have mild effect on the acrosomal structure in testis and more severe defective acrosome occurred due to mechanical shear during the passage of sperm through the epididymis and epididymal storage. Thus, the effects of *Ccdc28a* deletion on the epididymal sperm was severe than that on testis sperm.

In the proteomic analysis *Ccdc28a*^+/+^ and *Ccdc28a*^−/−^ cauda epididymis, MS-based proteomics analysis and Gene Ontology (GO) analysis showed that the expression profile for spermatogenesis changes significantly, wherein dozens of proteins required for spermatogenesis (such as RPL10L, ATP1A4, YBX2, SPATA20, PSMA8, ACTL7A and SPACA1) [24–29] and for the epididymal maturation (such as SPAM1, CLGN, LDHC, SORD and GSK3A) [6, 30–33] were significantly reduced. Knockout of these corresponding genes results in significant phenotypes in mice. For example, spermatocytes are finally arrested in M-phase by disruption of PSMA8 in mice, leading to abnormal spermatogenesis and male infertility [34]_._ Loss of SPACA1 and ACTL7A induce abnormal acrosome formation in spermatogenesis and male infertility [12, 29]. In addition, SPAM1, a marker of sperm maturation, plays an important role in epididymal sperm maturation processes due to enabling the sperm to penetrate the cumulus of oocytes via hyaluronidase activity [35]. Finally, deletion of lactate dehydrogenase type C (LDHC) disrupts ATP production and motility in sperm causing a failure in epididymal maturation male fertility [32]. These findings all suggest that CCDC28A is essential for male fertility.

Overall, we found that loss of CCDC28A can lead to male infertility and that ICSI can overcome the infertile phenotype of *Ccdc28a*^–/–^ mice. Our work demonstrated that CCDC28A was essential for male fertility by affecting the sperm morphology and motility. CCDC28A deletion caused a decreased SPACA1 and GSK3A protein levels in epididymal sperm which might indicate CCDC28A function by affecting the SPACA1 and GSK3A. As SPACA1 is located to acrosome, the loss of it in cauda might be also due to the loss of acrosome in cauda spermatozoa. However, due to lack of effective CCDC28A immunostaining antibody, the location of CCDC28A was unclear which needed to be further confirmed. This study highlights the important role of CCDC28A in regulating sperm morphology and motility which could explain the phenotype of patient with teratozoospermia.

## Materials and methods

### Generation of *Ccdc28a* knockout mice using CRISPR/Cas9

*Ccdc28a*^−/−^ mice were generated using the CRISPR/Cas9-mediated gene editing technology as previously described. To generate *Ccdc28a*^−/−^ mice, Cas9 protein was complexed with single-guide RNA 1 (CCA GGA CCC GGT GGA CCA ACT GG) and single-guide RNA 2 (GGT AGC TGG AAT GTT ACG GTA GG) targeting exon 3 and delivered into B6D2F1 (C57BL/6N × DBA/2) mouse zygotes. The surviving injected zygotes were transferred to the oviducts of pseudopregnant ICR female mice. The *Ccdc28a* knockout mouse model was confirmed by genomic PCR and sequence analysis. The PCR primers for genotyping were as follows: forward primer (F1), 5″-GTT GTA GCC CTC AGT GCC CTT G-3″, forward primer (F2), 5″-CTA TCA GCT TTG GTC CAG GTT CAC-3″; and reverse primer (R1), 5″-ATA AAC AGG GTG TCA GGG CAT GTG-3″. Primers F1 and R1 were used for the mutant, producing a 511 bp band. Primers F2 and R1 were for the wild type, producing a 476 bp band. Mouse breeding conditions and treatment methods in this experiment according to Animal Ethics Committee of Fudan University Shanghai Medical School (Shanghai, China).

### Semen parameters and sperm motility analysis

*Ccdc28a*^+/+^ and *Ccdc28a*^−/−^ adult male mice at 8–10 weeks of age were sacrificed by cervical dislocation. Mature sperm were collected from the cauda epididymis and then incubated in human tubal fluid (HTF) medium at 37 °C in a 5% CO_2_ humidified incubator for 45 min and assessed using computer-assisted sperm analysis (CASA).

### Reverse-transcription quantitative polymerase chain reaction (qRT-PCR)

Total RNA was extracted from mouse tissues using an miRNeasy Micro Kit (Qiagen, Germany), and cDNA was synthesized using a PrimeScript RT Reagent Kit with gDNA Eraser (Takara Biotechnology, China). The primers used for amplification of *Ccdc28a* were forward primer, 5′-CTG CAG GCG TTT GGA AAT GA-3′ and reverse primer, 5′-ACT GGC TGC TTT CCG CTT AT-3′. The primers for the *Gapdh* control were forward primer, 5′-ACC CTT AAG AGG GAT GCT GC-3′ and reverse primer, CCC AAT ACG GCC AAA TCC GT-3′. qRT-PCR was then performed with a SYBR Green Premix Ex Taq kit (Takara Biotechnology, China) according to the manufacturer's instructions on a Prism7300 machine (Applied Biosystems, USA).

### Western blotting

Testes or cauda epididymis from adult mice (8–10 weeks old) were homogenized in urea lysis buffer (7 M urea, 2 M thiourea, 65 mM DTT (dithiothreitol) with 1% v/v protease inhibitor cocktail) and subsequently disrupted by sonication. The samples were denatured in sodium dodecyl sulphate (SDS) loading buffer and separated by SDS- polyacrylamide gel electrophoresis (PAGE). The separated proteins were then transferred onto a nitrocellulose filter membrane and blocked with 5% fat-free milk in PBS with Tween-20. Antibodies included rabbit polyclonal anti-CCDC28A (Proteintech, 26434-AP-1, 1:1000 dilution), anti-SPACA1 (Abcam, 1:1000 dilution), rabbit polyclonal anti-GSK3A (ABclonal, 19721–1-AP, 1:1000 dilution), and anti-a-tubulin (Abcam, 1:3000 dilution). We used goat anti-rabbit IgG and goat anti-mouse IgG conjugated horseradish peroxidase at a 1:10,000 dilution to detect the primary antibodies. An ECL kit was used for chemiluminescence detection.

### IVF and ICSI

C57BL/6 female mice at 10 weeks of age were superovulated by injection of 7.5 IU of pregnant mare serum gonadotropin (PMSG) followed 48 h later by 7.5 IU of human chorionic gonadotropin (hCG). After 13 h, sperm were isolated from the cauda epididymis of *Ccdc28a*^+*/*+^ and *Ccdc28a*^*–/–*^ mice and capacitated for 50 min using HTF solution at 37 °C with 5% CO_2_. Cumulus-oocyte complexes were released from the ampulla of the uterine tube and transferred into a new HTF drop for fertilization at 37 °C in a humidified atmosphere of 5% CO_2_. After 6 h, 2PN zygotes were transferred to liquid drops of KSOM medium at 37 °C in 5% CO_2_ for blastocyst culture. Two-cell embryos were counted at 24 h postfertilization under a microscope. For ICSI, the steps of superovulation were similar to IVF. Female mice were superovulated via the injection of 7.5 IU PMSG and 75 IU hCG given 48 h apart. After 13 h, sperm were isolated from the cauda epididymis of *Ccdc28a*^+/+^ and *Ccdc28a*^–/–^ mice and were sonicated to detach the heads from the tails and then injected into B6D2F1 mouse oocytes that had been obtained from superovulated females by a Piezo-driven pipette. After injection, the oocytes were cultured in KSOM medium at 37 °C under 5% CO_2_ for subsequent development.

### TEM and scanning electron microscopy

Testicular tissue and cauda epididymal sperm obtained from *Ccdc28a*^+/+^ and *Ccdc28a*^–/–^ mice were fixed with 2.5% (vol/vol) glutaraldehyde for 2 h. Samples were washed four times with 0.1 M phosphate buffer and were fixed with 1% osmic acid. After washing a few times, the samples were dehydrated through an ethanol series and then embedded in plastic resin. Ultrathin sections (60 nm) were cut and counterstained with uranyl acetate and lead citrate. Images were acquired using a transmission electron microscope (Philips CM-120, Netherlands).

For scanning electron microscopy, sperm from *Ccdc28a*^+/+^ and *Ccdc28a*^–/–^ mice were fixed with 2.5% (vol/vol) glutaraldehyde and 1% osmic acid. After washing three times, the samples were dehydrated through a graded ethanol series and dried with a CO_2_ critical-point dryer. The dried specimens were mounted on aluminum stubs and coated with gold for 3 min. Images were obtained from an SU801 scanning electron microscope (HITACHI SU8010, Japan).

### PAS staining, TUNEL assay and Immunofluorescence staining

Testes were collected from *Ccdc28a*^+/+^ and *Ccdc28a*^–/–^ adult male mice and fixed with 4% Bouins solution overnight at 4 °C. The fixed tissues were then embedded in paraffin, sectioned at 5 μm, dewaxed, and rehydrated through a graded ethanol series. PAS staining was carried out in accordance with the manufacturer’s protocols (Beijing Solarbio Science & Technology). Briefly, the sections were incubated in periodic acid for 5 min at room temperature and rinsed in distilled water. They were then stained with Schiff’s solution for 10 min at room temperature in the dark. The sections were stained with hematoxylin for 5 min at room temperature and put in 1% hydrochloric acid alcohol (6 dips). After dehydration with graded alcohol (70, 90, and 100%), the sections were sequentially immersed in xylene twice for 10 min both times. Images were obtained by light microscopy. The TUNEL assay of the tissue sections was performed as described previously [36]. For immunofluorescence, tissue sections were blocked in antibody dilution buffer (3% BSA) for 60 min, followed by overnight incubation at 4 °C with primary antibodies against SPACA1 (Abcam, 1:100 dilution). After washing with PBST four times, sections were incubated with secondary antibodies (Thermo Fisher, Alexa Fluor 555 goat anti-rabbit IgG, 1:500), Hoechst 33342 solution (Abcam, 1:500 dilution) and lectin PNA (Sigma, 1:500 dilution) at 37 °C for 1 h. Finally, visualized using laser scanning confocal microscopy (LSM880, Zeiss).

### In situ hybridization

Anti-sense *Ccdc28a* mRNA probes were designed and purchased from biosune (Shanghai, China) and Non-radioactive detection of mRNA in sections of the mouse testis and epididymis was performed as previously described [37]. Fresh mouse testes and epididymis fixed in 4% paraformaldehyde at 4 °C for 24 h, and then were incubated in 30% sucrose overnight at 4 °C. Next, sectioned at 4 μm using a cryostat (Leica) were cut from mouse testis/epididymis fresh-frozen tissue on a cryostat and was pre-hybridized for 2 h and hybridized with the CY3-labeled anti-sense *Ccdc28a* mRNA probes at 55 °C overnight. After extensive washing (at 50 °C), the sections were Hoechst 33342 solution (Abcam, 1:500 dilution) and lectin PNA (Sigma, 1:500 dilution) for 1 h, and then were visualized using laser scanning confocal microscopy (LSM880, Zeiss).

### Protein extraction and quantitative proteomic analysis

The cauda epididymis from 2-month-old *Ccdc28a*^+/+^ and *Ccdc28a*^−/−^ male mice was used for proteomic analysis. Briefly, the cauda epididymis was homogenized with urea lysis buffer (7 M urea, 2 M thiourea, and 65 mM dithiothreitol) supplemented with 1% v/v protease inhibitor cocktail and disrupted by sonication. Protein lysate was centrifuged at 12,000×*g* at 4 °C for 25 min, and the supernatant protein concentration was measured using the bicinchoninic acid method. The concentration and total amount of protein was equalized for all samples. The samples were then digested with trypsin and analyzed on an Orbitrap Exploris 480 mass spectrometer (Thermo Fisher Scientific) coupled to an EASY-nLC 1000 nanoflow HPLC (Thermo Fisher Scientific). The raw MS data were searched against the mouse protein database from UniprotKB, and the cutoff of the global false discovery rate for peptide and protein identifications was adjusted to < 1%. Only proteins assigned at least with three unique peptides in at least two replicates were accepted. In addition, the proteins with fold change values above 1.5 or below 0.70 were deemed to be significantly differentially expressed (P < 0.05).

### Coimmunoprecipitation

Tissue extracts were homogenized in cold NP-40 lysis buffer (50 mM Tris, 150 mM NaCl, 0.5% NP-40, pH 7.5) containing 1% protease inhibitor cocktail (Bimake). The lysates were centrifuged at 15,000×*g* at 4 °C for 20 min, and the supernatant was divided into two groups—one group was treated with 2 μg anti-GSK3A antibody (ABclonal, 17355-1-AP) or anti-SPACA1 antibody (Abcam, ab191843), and the other group (negative control) was treated with 2 μg rabbit IgG nonspecific antibody (Abmart). After incubation overnight at 4 °C with gentle rotation, the prepared lysis complexes were incubated with 13 μl Protein A/G-conjugated beads for 2 h at 4 °C. The agarose beads were washed four times with cold NP-40 lysis buffer. Finally, the beads were resuspended in 2 × SDS loading buffer and boiled for 10 min and then subjected to Western blotting.

### Statistical analysis

We performed all experiments at least three times independently. Statistical significance was assessed by two-tailed Student’s t-test or one-way ANOVA in GraphPad Prism 8, and P < 0.05 were considered statistically significant. Brown-Forsythe test was performed to compare the homogeneity of variances. If the data had homogeneous variances, unpaired t test was used for two group comparisons of means. If the data did not have similar variances, unpaired t test with Welch’s correction was performed.

### Supplementary Information

Below is the link to the electronic supplementary material.

Supplementary file1 (DOCX 1811 KB)

Supplementary file2 (DOCX 41 KB)

## Data Availability

All data analyzed during this study are included in in the main text or the supplementary materials.
