# Supplementary material for: CCDC28A deficiency causes sperm head defects, reduced sperm motility and male infertility in mice
Source: Cell Mol Life Sci. 2024 Apr 10;81(1):174. doi: 10.1007/s00018-024-05184-5 (PMC11006775; doi:10.1007/s00018-024-05184-5)
Supplement: Supplementary file 1 — Supplementary file1 (DOCX 1811 KB) [file 18_2024_5184_MOESM1_ESM.docx]

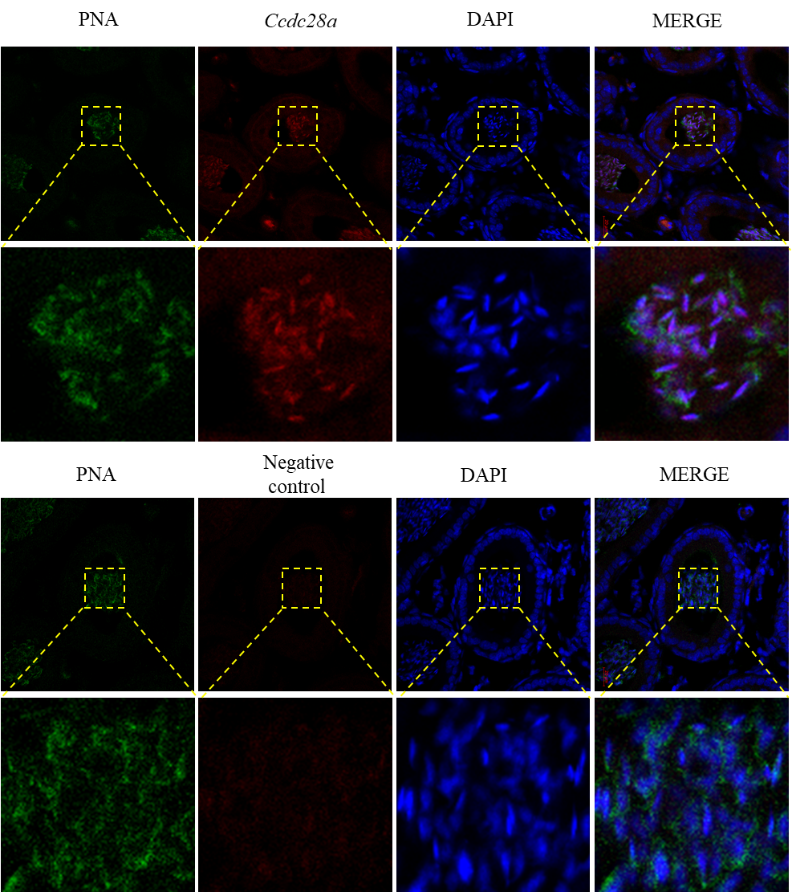


**Fig S1.** **In situ hybridization for *Ccdc28a* mRNA on** **epididymis sections of sexually mature mice.** Representative images of mouse epididymis epididymis sections stained for PNA (green), acrosome; *Ccdc28a* mRNA (red); Hoechst (blue), nucleus. Scale bar=20 µm.


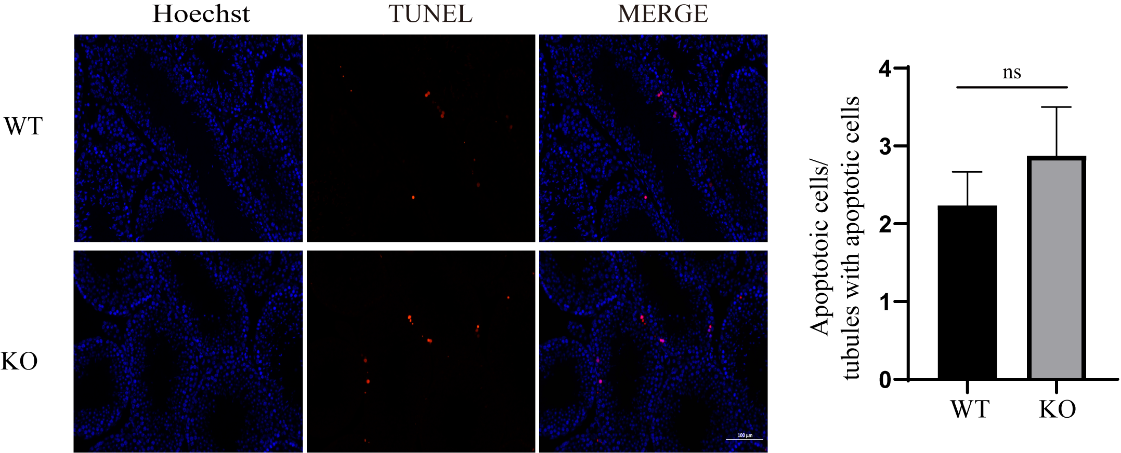


**Fig S2. Terminal deoxynucleotidyl transferase nick end labeling (TUNEL) staining showed apoptotic cells in the testes of *Ccdc28a*^+/+^ and *Ccdc28a*^–/–^ mice.** The red signals indicate the apoptotic cells, ns, non-significant. Scale bar = 100 μm.


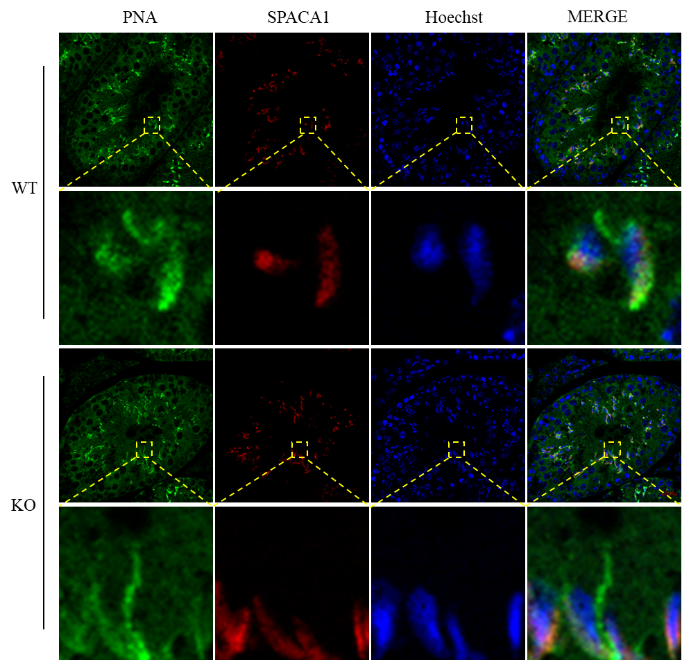


**Fig S3. CCDC28A-KO mice show no differences in SPACA1 localization compared to the control mice.** Immunofluorescence staining for the acrosome and SPACA1 during spermatogenesis

using testis sections. PNA (green), acrosome; Hoechst (blue), nucleus; SPACA1 (red). Scale bar=20 µm.
