# Supplementary material for: CCDC28A deficiency causes sperm head defects, reduced sperm motility and male infertility in mice
Source: Cell Mol Life Sci. 2024 Apr 10;81(1):174. doi: 10.1007/s00018-024-05184-5 (PMC11006775; doi:10.1007/s00018-024-05184-5)
Supplement: Supplementary file 2 — Supplementary file2 (DOCX 41 KB) [file 18_2024_5184_MOESM2_ESM.docx]

Table S1. Differentially expressed proteins in *Ccdc28a*^–/–^ mouse testes compared to *Ccdc28a* ^+/+^ mouse testes.

| **Gene** | **log2FoldChange** | **pvalue** | **Gene** | **log2FoldChange** | **pvalue** |
| --- | --- | --- | --- | --- | --- |
| *Rab2b* | -4.778051098 | 1.87E-05 | *Ace* | -0.598861337 | 0.038591513 |
| *Mns1* | -3.744676947 | 4.44E-22 | *Adsl* | 0.603910127 | 0.049684292 |
| *Fam71b* | -2.964583733 | 1.58E-09 | *Hspa4* | 0.616099742 | 0.039823223 |
| *Atp1a3* | -2.893578192 | 6.13E-10 | *Rps8* | 0.620900765 | 0.040013605 |
| *Tex101* | -2.761584844 | 1.46E-19 | *Pdcd6* | 0.626900029 | 0.038600876 |
| *Dynll2* | -2.280242625 | 1.29E-12 | *Rsu1* | 0.629148216 | 0.043312403 |
| *Vamp3* | -2.042573674 | 8.26E-07 | *Stip1* | 0.632269402 | 0.026247067 |
| *Tex55* | -1.97257992 | 0.00237397 | *Tbca* | 0.633860824 | 0.042701904 |
| *Dpep3* | -1.895055128 | 1.70E-06 | *Capza1* | 0.642067252 | 0.042608008 |
| *Prss39* | -1.835247436 | 0.000224515 | *Nap1l1* | 0.647971487 | 0.035436782 |
| *Glipr1l1* | -1.802176976 | 9.10E-05 | *Kpnb1* | 0.655823934 | 0.037767764 |
| *Actl7a* | -1.739729545 | 7.58E-05 | *Col6a2* | 0.670093789 | 0.018782721 |
| *Oxct2a* | -1.690497499 | 0.004050274 | *Ecm1* | 0.674269457 | 0.033834919 |
| *Spink2* | -1.681701791 | 1.73E-08 | *Ywhaq* | 0.677756686 | 0.027940462 |
| *Ranbp17* | -1.676685392 | 2.49E-06 | *Sf3b5* | 0.68227881 | 0.0296826 |
| *Hk1* | -1.598927853 | 5.31E-05 | *Pcmt1* | 0.69023868 | 0.031381056 |
| *Il4i1* | -1.597300161 | 8.52E-07 | *Plin4* | 0.71069275 | 0.048415232 |
| *Spata48* | -1.588445534 | 0.00019643 | *Hars1* | 0.712657921 | 0.031224449 |
| *Spam1* | -1.562031951 | 0.001549849 | *Ltf* | 0.713594883 | 0.033783888 |
| *Sptb* | -1.557698473 | 0.005263465 | *Lgals1* | 0.720500437 | 0.014476189 |
| *Ribc1* | -1.542196553 | 0.000320685 | *Cnn2* | 0.721180813 | 0.040105628 |
| *G6pd2* | -1.516269271 | 0.001981861 | *Coro1c* | 0.740763736 | 0.025318713 |
| *Samd15* | -1.51395727 | 0.010393446 | *Tsn* | 0.755162101 | 0.024919372 |
| *Pgk2* | -1.504261726 | 0.000242707 | *Cog3* | 0.755896307 | 0.034271257 |
| *Sord* | -1.502070918 | 1.53E-05 | *Ppp5c* | 0.756486308 | 0.023348875 |
| *Zp3r* | -1.462352563 | 0.000369862 | *Dcaf7* | 0.756689584 | 0.043987468 |
| *Zpbp2* | -1.441690981 | 0.001015106 | *Nid2* | 0.759075303 | 0.018487869 |
| *Cfap44* | -1.402499279 | 0.033956631 | *Vim* | 0.777200845 | 0.018335847 |
| *Cgn* | -1.385531308 | 0.028840348 | *Psap* | 0.779888782 | 0.016287398 |
| *Pdzk1* | -1.364881721 | 0.001137131 | *Tsnax* | 0.789256194 | 0.021553169 |
| *Dydc1* | -1.363639496 | 0.001027999 | *Xdh* | 0.793637721 | 0.03753232 |
| *Lypd4* | -1.355064527 | 0.001948657 | *Aimp2* | 0.795293337 | 0.027028352 |
| *Lmntd2* | -1.342349639 | 0.001342936 | *Lgals3bp* | 0.802323265 | 0.007883176 |
| *Izumo4* | -1.337341666 | 0.004850839 | *Rnase9* | 0.80808667 | 0.045182079 |
| *Igbp1b* | -1.334372735 | 0.013999903 | *Cp* | 0.811951267 | 0.046389463 |
| *Nsun4* | -1.306890726 | 0.016968809 | *Aamdc* | 0.828973855 | 0.018161989 |
| *Csnk1g1* | -1.296550997 | 0.004676361 | *Azgp1* | 0.839573118 | 0.041642385 |
| *Csnk1g3* | -1.296550997 | 0.004676361 | *Fbln5* | 0.848862317 | 0.018593034 |
| *Gas8* | -1.295560892 | 0.001278676 | *Ehd1* | 0.849639588 | 0.048447268 |
| *Spata18* | -1.292633613 | 0.007521257 | *Abce1* | 0.851334792 | 0.021831528 |
| *Tmem30a* | -1.291650295 | 0.000104016 | *Sdf4* | 0.854239589 | 0.008590786 |
| *Ubxn11* | -1.288094936 | 0.005929859 | *Sephs2* | 0.859160231 | 0.043814774 |
| *Hdhd2* | -1.278648441 | 0.002106068 | *Csde1* | 0.860969972 | 0.022470416 |
| *Fam205c* | -1.273654591 | 0.00076331 | *Efhd2* | 0.861587007 | 0.008757009 |
| *Atp5if1* | -1.264844193 | 0.005917489 | *Lpcat3* | 0.87254356 | 0.010422373 |
| *Prss46* | -1.263822662 | 0.000539258 | *Man1a1* | 0.885939549 | 0.037689435 |
| *Acr* | -1.238342492 | 0.005126895 | *Aspscr1* | 0.886054753 | 0.039060444 |
| *Ment* | -1.238152107 | 0.008922341 | *Hook1* | 0.886193907 | 0.017652785 |
| *Man2b2* | -1.220400932 | 0.000722114 | *Dctn1* | 0.892231532 | 0.022775293 |
| *Clmn* | -1.212766389 | 0.025462538 | *Gspt1* | 0.892772342 | 0.003706837 |
| *Csnk1g2* | -1.194453646 | 0.004096088 | *Plekho2* | 0.895506173 | 0.048500998 |
| *Pebp4* | -1.193243303 | 0.004450113 | *Sec63* | 0.900953089 | 0.018063009 |
| *Ak1* | -1.19270169 | 0.011866501 | *Dpysl3* | 0.904479797 | 0.021052142 |
| *Rsph3a* | -1.176619838 | 0.02137273 | *Lgmn* | 0.904723385 | 0.011797012 |
| *Rsph3b* | -1.176619838 | 0.02137273 | *Usp4* | 0.905181978 | 0.02560465 |
| *Rab4b* | -1.157151236 | 0.004321264 | *Ero1b* | 0.906326251 | 0.030220372 |
| *Cabyr* | -1.145229102 | 0.005504284 | *Acyp1* | 0.90691831 | 0.003306873 |
| *Fscb* | -1.144312038 | 0.008236687 | *Cdv3* | 0.907937643 | 0.003182604 |
| *Bpi* | -1.144042933 | 0.003919964 | *Rangap1* | 0.909773158 | 0.020079725 |
| *Pgam2* | -1.143945858 | 0.00149965 | *Rpl35* | 0.928207693 | 0.017074315 |
| *Fabp9* | -1.131919643 | 0.016335448 | *Tln2* | 0.935670778 | 0.027117796 |
| *Acrv1* | -1.13181801 | 0.006122841 | *Hspa4l* | 0.942396881 | 0.010931585 |
| *Thegl* | -1.125435711 | 0.017546126 | *Nt5c3b* | 0.946973665 | 0.007390819 |
| *Phospho1* | -1.117870635 | 0.026140168 | *Ftl1* | 0.951741828 | 0.028931707 |
| *Acsbg2* | -1.111189364 | 0.027534407 | *Ftl2* | 0.951741828 | 0.028931707 |
| *Slc2a5* | -1.107429936 | 0.014132393 | *Hnrnpul1* | 0.954861287 | 0.011450159 |
| *Atp1a4* | -1.098613665 | 0.002062931 | *C9* | 0.960679109 | 0.016375343 |
| *Dynlrb2* | -1.096879019 | 0.014388632 | *Ap2b1* | 0.964216055 | 0.002467523 |
| *Bspry* | -1.094499954 | 0.009322655 | *Sh3gl1* | 0.965531806 | 0.009121175 |
| *Spesp1* | -1.088393997 | 0.022909312 | *Herc4* | 0.975805695 | 0.017158436 |
| *Crat* | -1.086560038 | 0.024348429 | *Serpine2* | 0.976986131 | 0.020467234 |
| *Larp1* | -1.084798061 | 0.012720466 | *Eif4e* | 0.986177112 | 0.003491427 |
| *Dbil5* | -1.07710969 | 0.003966497 | *Lancl2* | 1.000866452 | 0.023275247 |
| *Psma8* | -1.066998193 | 0.006328199 | *Adipoq* | 1.004063939 | 0.025999121 |
| *Ppp1r2* | -1.066117762 | 0.000287247 | *Actbl2* | 1.012523927 | 0.040872072 |
| *Prpsap2* | -1.062158196 | 0.029929499 | *Qsox1* | 1.014578765 | 0.003049866 |
| *Ndufs4* | -1.060367728 | 0.012745311 | *Cpn1* | 1.036680389 | 0.017062786 |
| *Ptchd3* | -1.060292438 | 0.015630289 | *H1-0* | 1.079955254 | 0.004029951 |
| *Thnsl1* | -1.059718935 | 0.010375881 | *Hagh* | 1.081104625 | 0.006132761 |
| *Cpa5* | -1.041566 | 0.002499436 | *Sh3glb1* | 1.099088897 | 0.005284377 |
| *Gstm5* | -1.041421974 | 0.000910659 | *Spag9* | 1.105180917 | 0.017299696 |
| *Prcp* | -1.04129611 | 0.015722182 | *Arl3* | 1.106574205 | 0.00091027 |
| *Pfkp* | -1.027327567 | 0.005191912 | *Armc12* | 1.107912732 | 0.008589524 |
| *Tex33* | -1.021433126 | 0.035508164 | *Ube2v2* | 1.112345275 | 0.005282879 |
| *Mmel1* | -1.018311402 | 0.018304059 | *Adgre5* | 1.119538384 | 0.000443061 |
| *Ppp1r7* | -1.015932078 | 0.002866999 | *Ovos* | 1.123959701 | 0.009718106 |
| *Arsa* | -1.006730443 | 0.016964157 | *Thop1* | 1.124164758 | 0.001651787 |
| *Tssk4* | -1.006577236 | 0.030176881 | *Ngly1* | 1.129066014 | 0.020679861 |
| *Csnk1a1* | -1.00478198 | 0.043089745 | *Cog5* | 1.129306587 | 0.01360417 |
| *Htra2* | -0.99438478 | 0.013772232 | *Memo1* | 1.131159657 | 0.022891775 |
| *Prkaca* | -0.993535259 | 0.006943019 | *Rai14* | 1.151391586 | 0.004369528 |
| *Ca2* | -0.992545221 | 0.023716588 | *Cap2* | 1.163346986 | 0.003495769 |
| *Prss21* | -0.992398735 | 0.005648416 | *Ggta1* | 1.199224443 | 3.55E-05 |
| *Acrbp* | -0.991278395 | 0.036348338 | *Stmn1* | 1.204309988 | 0.003981248 |
| *Mroh2b* | -0.990296696 | 0.01355376 | *Lancl1* | 1.210677101 | 0.003089435 |
| *Cyb5r2* | -0.989627306 | 0.00954877 | *Mycbp2* | 1.213213767 | 0.00157927 |
| *Banf2* | -0.985963039 | 0.004714696 | *Tsks* | 1.214616583 | 0.001709756 |
| *Nt5c1b* | -0.983371901 | 0.008872751 | *Ybx3* | 1.220504479 | 0.000222506 |
| *Cdc37l1* | -0.977595451 | 0.006185632 | *Akap1* | 1.229851434 | 0.001594608 |
| *Prkar2a* | -0.968297503 | 0.013454384 | *Hmox2* | 1.231416591 | 0.001157006 |
| *Art3* | -0.960321614 | 0.003231578 | *Eif2s3y* | 1.247466032 | 0.009878619 |
| *Bpgm* | -0.95558172 | 0.024581755 | *Akr1b8* | 1.25433514 | 0.003199881 |
| *Fscn3* | -0.951184995 | 0.008435562 | *Carhsp1* | 1.269576824 | 0.000889681 |
| *Stx12* | -0.947652013 | 0.02629831 | *Lztfl1* | 1.274553019 | 0.000385477 |
| *Ak2* | -0.946789378 | 0.011978206 | *Cul5* | 1.312498974 | 0.001165896 |
| *Cyct* | -0.934260151 | 0.018705145 | *Serping1* | 1.317762669 | 0.000432774 |
| *Isoc2a* | -0.929777395 | 0.008687172 | *Mup2* | 1.318580005 | 0.014071624 |
| *Spata7* | -0.916631615 | 0.029578633 | *Col1a2* | 1.363420048 | 0.004860259 |
| *Gsk3a* | -0.896575487 | 0.004622862 | *Mprip* | 1.368628852 | 0.006283431 |
| *Atrx* | -0.894015899 | 0.036320731 | *Clgn* | 1.382406024 | 0.000988035 |
| *Psmd11* | -0.88072593 | 0.003177246 | *Hsp90aa1* | 1.42558884 | 1.14E-06 |
| *Kpna3* | -0.880040269 | 0.014335468 | *Eif4a2* | 1.428629761 | 8.29E-07 |
| *Zpbp* | -0.874993608 | 0.033836516 | *Rpl10l* | 1.453634221 | 0.032833173 |
| *Psmc4* | -0.873076406 | 0.002483879 | *Creld2* | 1.47039216 | 2.41E-05 |
| *Ckmt1* | -0.872598768 | 0.023231423 | *Agt* | 1.48003404 | 9.13E-05 |
| *Rad23a* | -0.872172157 | 0.011771683 | *Cpne1* | 1.560677827 | 0.000556399 |
| *Ndufb9* | -0.867166896 | 0.001700338 | *Cma1* | 1.585098621 | 0.002761596 |
| *Tppp2* | -0.861239672 | 0.019643868 | *Alg2* | 1.598422047 | 0.004253008 |
| *Ldhc* | -0.858926093 | 0.015310111 | *Ppid* | 1.612657328 | 0.002175145 |
| *Dnpep* | -0.857601967 | 0.016898633 | *Glb1l3* | 1.655340505 | 6.25E-05 |
| *Ndufa8* | -0.853743315 | 0.004999885 | *Serpinf1* | 1.682259577 | 0.026446704 |
| *Eqtn* | -0.851682109 | 0.014119512 | *Pdilt* | 1.722127987 | 3.47E-05 |
| *Nckap1* | -0.849794798 | 0.021850188 | *Pgp* | 1.727967742 | 2.46E-07 |
| *Timm50* | -0.839013283 | 0.011820802 | *Cacybp* | 1.732948303 | 4.75E-07 |
| *Tekt3* | -0.835987093 | 0.04764516 | *Septin6* | 1.747968143 | 0.000618418 |
| *Atp1b3* | -0.833400922 | 0.02830877 | *Ociad2* | 1.752958384 | 0.001323834 |
| *Gpi* | -0.820082192 | 0.011720432 | *Hspa2* | 1.759544405 | 7.95E-08 |
| *Ndufa7* | -0.814264442 | 0.00932062 | *Pbp2* | 1.801157769 | 4.35E-05 |
| *Apool* | -0.813897856 | 0.018712996 | *Ipo5* | 1.802230785 | 1.42E-05 |
| *Cox6b2* | -0.807694479 | 0.037009082 | *Mup1* | 1.81307306 | 5.89E-05 |
| *Auh* | -0.80612192 | 0.046920922 | *Nrdc* | 1.845411663 | 4.35E-05 |
| *Cox5a* | -0.797775647 | 0.045637489 | *Dnase1l2* | 1.887142565 | 1.94E-05 |
| *Kif9* | -0.79094589 | 0.025943864 | *Tcp11* | 1.892350702 | 0.000167726 |
| *Spaca1* | -0.788646105 | 0.04623475 | *Prdx4* | 1.892614606 | 8.29E-08 |
| *Tkfc* | -0.769789104 | 0.032695062 | *Mex3d* | 1.91751085 | 0.000626305 |
| *Sfxn2* | -0.767740289 | 0.032796695 | *Cfap36* | 1.92366831 | 0.000592036 |
| *Acat1* | -0.758793599 | 0.041141007 | *Lcn2* | 1.964624773 | 4.79E-07 |
| *Uchl5* | -0.758346307 | 0.018754188 | *Tmx4* | 1.981450603 | 9.22E-07 |
| *Ccdc113* | -0.757597698 | 0.047830839 | *Cog4* | 2.006462997 | 2.83E-09 |
| *Nsf* | -0.755779039 | 0.032017996 | *Otub2* | 2.010326681 | 0.005719012 |
| *Sod2* | -0.730953022 | 0.009166828 | *Mmp7* | 2.038638641 | 3.08E-05 |
| *Mycbp* | -0.721381423 | 0.047834662 | *Spata20* | 2.04949756 | 0.00167069 |
| *Uqcrfs1* | -0.719625804 | 0.017850988 | *Fxr1* | 2.078358157 | 2.35E-07 |
| *Ppp3cc* | -0.712999616 | 0.041623382 | *Txnrd3* | 2.089238622 | 1.60E-09 |
| *Por* | -0.704535058 | 0.024196645 | *H1-3* | 2.150280939 | 0.019185428 |
| *Ace3* | -0.704176842 | 0.031574701 | *Rnase13* | 2.151452547 | 1.88E-07 |
| *Fam166c* | -0.702618428 | 0.020209162 | *Rnase10* | 2.18435749 | 1.04E-06 |
| *Slc27a2* | -0.701600212 | 0.020357025 | *Clu* | 2.192857866 | 8.07E-08 |
| *Psmd4* | -0.699998323 | 0.015333816 | *Diablo* | 2.210193014 | 8.74E-07 |
| *Akr7a2* | -0.684796237 | 0.026906707 | *Fabp4* | 2.228422666 | 3.15E-06 |
| *Cryz* | -0.681734219 | 0.036695446 | *Plau* | 2.276012217 | 0.00023702 |
| *Uqcrq* | -0.672175002 | 0.044804789 | *Actg2* | 2.309569106 | 1.97E-05 |
| *Hsd17b12* | -0.664134276 | 0.026959312 | *Ca3* | 2.484206634 | 9.73E-05 |
| *Hrg* | -0.655397429 | 0.039428058 | *Ugt1a6* | 2.501845901 | 0.008545491 |
| *Glb1l* | -0.651001743 | 0.035232066 | *Tomm34* | 2.562019044 | 2.91E-18 |
| *Slk* | -0.650996567 | 0.044277964 | *Spint4* | 2.575744618 | 3.91E-07 |
| *Psmd1* | -0.648932245 | 0.028154298 | *Cby2* | 2.615822468 | 3.78E-13 |
| *Psmc1* | -0.644830586 | 0.037652989 | *Clca3a1* | 2.668299234 | 1.29E-08 |
| *Aldoa* | -0.639842142 | 0.039220365 | *Ybx2* | 2.736869761 | 2.06E-09 |
| *Lpp* | -0.639680821 | 0.023504429 | *Cabs1* | 2.807650294 | 9.31E-21 |
| *Eno1* | -0.632390985 | 0.02370854 | *Ddx3y* | 2.924347545 | 0.004179594 |
| *Mix23* | -0.628969231 | 0.033267837 | *Mfge8* | 3.582248433 | 2.96E-16 |
| *Park7* | -0.61053611 | 0.022336624 |  |  |  |

Note: The proteins with fold change values above 1.5 or below 0.7 were deemed to be significantly differentially expressed (P < 0.05), and a total of 161 upregulated and 164 downregulated proteins were found.
